# The family physician as a primary care consultant – the Mossel Bay experience

**DOI:** 10.4102/phcfm.v13i1.3061

**Published:** 2021-09-30

**Authors:** Klaus B. von Pressentin, Kartik S. Naidoo, Louis S. Jenkins, Johann Schoevers

**Affiliations:** 1 Division of Family Medicine, School of Public Health and Family Medicine, University of Cape Town, Cape Town, South Africa; 2 Department of Family and Emergency Medicine, Faculty of Medicine and Health Sciences, Stellenbosch University, Cape Town, South Africa; 3 Mossel Bay Hospital, Garden Route District, Western Cape Department of Health, Mossel Bay, South Africa; 4 George Hospital, Garden Route District, Western Cape Department of Health, George, South Africa; 5 Primary Health Care Directorate, Faculty of Health Sciences, University of Cape Town, Cape Town, South Africa

**Keywords:** family physicians, primary healthcare, district health system, primary care, consultant, South Africa

## Abstract

The South African family physician (FP) is an expert generalist who has a number of roles to strengthen the district health system. A research study on FPs in district hospitals has previously demonstrated an impact; however, more evidence on impact in primary health care (PHC) is needed. By serving as a consultant for the PHC team, the FP may improve access to care, capacitate team members, enhance comprehensiveness of care, and improve coordination and continuity of care. This report narrates the story of how one of the FPs at a rural district hospital recorded his experience of being a consultant to the PHC team and was able to self-audit the experience. A self-designed audit tool analysed 1000 patient consultations with the FP and enabled a reflection on the coronavirus disease 2019 (COVID-19)-related changes to the consultant role. There was a clear need for FPs to consult patients with complex multi-morbidity and multifaceted psychosocial aspects to their illness, in consultation with their team members. Patients were referred to them by medical officers, other specialists, family medicine registrars, allied healthcare professionals and nurse practitioners. The FP’s ability to strengthen the PHC service outside the district hospital may be enhanced by creating more FP posts at a subdistrict level to support high-quality, team-based primary care in line with the PHC policy directions.

## Background

The South African family physician (FP) is trained as an expert generalist to strengthen primary healthcare (PHC) in the district health system (DHS). Their roles include clinical governance, capacity building, clinical training and championing of community-orientated primary care.^1^ Serving as a consultant is a lesser described role, and more research studies are needed to understand the scope of this role within the PHC team.^2^ A previous research study documented the contribution of FPs to strengthening the South African DHS, particularly their value in the district hospital.^3^ More evidence is required on the contribution of FPs to PHC services.^2^ This short report describes the value added by FPs as consultants to the PHC services.

## Context

The Mossel Bay subdistrict forms part of the Garden Route District in the Western Cape province, South Africa and serves a population of 95 255 people.^4^ This subdistrict consists of a 90-bed district hospital, which covers a geographic area of 2000 km^2^ through five fixed PHC facilities, seven satellite clinics and four mobile clinics. The subdistrict team provides the full scope of the DHS, in partnership with the specialist departments at George regional hospital. The team of doctors include a clinical manager, a FP, two family medicine registrars, nine medical officers and two community service medical officers. Whilst these doctors are based at the district hospital to provide generalist inpatient and outpatient care, they also support the nurse-led primary care services in the subdistrict through outreach. The PHC clinics also receive other visiting healthcare professionals, including a dietician, a physiotherapist, an occupational therapist and a mental health nurse.

## Contribution of the family physicians

The first author started working in this subdistrict as a FP in September 2016. A key activity was to provide a consultant service to the PHC teams across various facilities. As the sole FP in the subdistrict, he had to ensure that his duties with regards to the six roles were shared between the district hospital and PHC facilities. In consultation with clinicians and managers, the FP reviewed his outreach service to PHC clinics as part of an effort to structure the working week better.

The FP reviewed referred patients at the hospital’s outpatient department (OPD) on Mondays (for referrals from PHC clinics and down-referrals from specialists at the regional hospital) and visited three PHC facilities on Tuesdays and Wednesdays (distances between the hospital and these PHC facilities ranged between 3 km and 25 km). Patients were referred to the FP by the PHC team members through an internal booking system at each facility. These PHC team members included the subdistrict medical officers, family medicine registrars and allied health colleagues (mainly the occupational therapist for attention deficit hyperactivity disorder work-up, as well as the clinical nurse practitioners for review of patients on second-line antiretroviral therapy [ART]). The referral criteria were not fixed, as it was necessary to ensure that the team members felt comfortable referring patients for clinical input whenever they needed assistance. The FP encouraged learning conversations with PHC team members to decide whether to see patients at his clinic or whether it would be more appropriate to refer directly to the regional hospital specialist service to ensure a streamlined journey through the healthcare system. The FP also encouraged clinical nurse practitioners to discuss possible FP referrals directly with the medical officer on-site at the PHC facility, as often the medical officer would be able to confirm the need for FP referral or initiate a quick phone call with the FP to discuss the most appropriate care plan strategy for the patient. This triaging step was necessary to ensure the efficient use of the FP outreach service. Referrals to the FP from the regional hospital specialists were made either during their outreach visits to the Mossel Bay subdistrict or from the regional hospital OPDs (referrals made via email communication or written letters).

The FP conducted a prospective audit to describe the nature of patients referred to him from the PHC team in order to ensure an efficient consultation service. The FP developed a simple Microsoft Excel tool, which he piloted during March 2018, to capture data on several indicators, such as the referring primary care provider, clinical problem, any special investigations, involvement of other providers (including allied health and other specialists), interventions (including counselling and motivational interviewing), and specialist code items prescribed (certain pharmaceutical items may not be prescribed by medical officers and required prescription by a specialist FP). Examples of these specialist code pharmaceutical items included disease-modifying antirheumatic drugs, carbimazole, amiodarone, atorvastatin, pregabalin, doxazosin and losartan.

**FIGURE 1:**
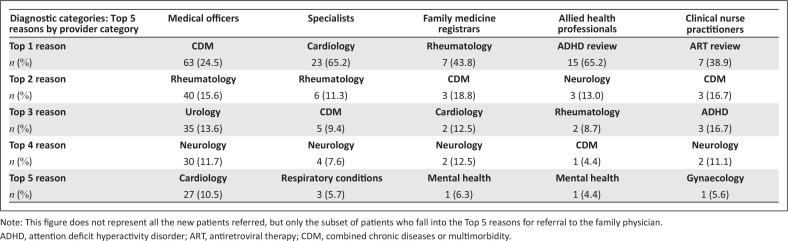
Top-five diagnostic categories of new patients referred by key providers (*n* = 371).

The FP gathered data over 18 months (April 2018 – August 2019) and used the data on the first 12 months to review and improve his practice. During this period, there was an increase in the proportion of patients who were new referrals from 33% during the first 12 months to 40% during the final 6 months, which reflected an enhanced access to the FP of patients newly referred by the PHC team members (Table 1). The FP continued the prospective audit until 1000 consultations were captured by January 2020. Figure 1 describes the top-five reasons for referral from different types of healthcare professionals to the FP.

**TABLE 1:** Description of family physician primary care consultations.

Variables	*n*	%
**New vs. follow-up patients (*n* = 1000)**		
Newly referred patients	371	37.1
Follow-up consultations	629	62.9
**Providers referring new patients to the FP (*n* = 371)**		
Medical officers (based in the subdistrict)	257	69.3
Specialists (regional hospital)	53	14.3
Family medicine registrars	16	4.3
Allied health professionals	23	6.2
Clinical nurse practitioners	18	4.8
Other sources	4	1.1
**Top-five diagnostic categories encountered during follow-up consultations (*n*= 629)**		
Combined chronic diseases or multimorbidity	191	30.4
Attention deficit hyperactivity disorder	109	17.3
Rheumatology	86	13.7
Neurology	43	6.8
Cardiology	40	6.4

FP, family physician.

## Contribution of intervention towards strengthening the district health system

From the findings shown in Figure 1, a few key diagnostic categories and referral scenarios may be identified. The integrated care pathway of children with suspected or confirmed attention deficit hyperactivity disorder (ADHD) needs to be highlighted, as the FP worked closely with the multidisciplinary team in consultation with the paediatrician to ensure accurate work-up, diagnosis and interdisciplinary care, including appropriate access to methylphenidate, a specialist prescription item. Referrals from the clinical nurse practitioners for review by the FP of patients on second-line ART mainly took place at the one of the PHC facilities, which is located furthest from the district hospital and does not receive visits from the medical officer specialised in HIV care. The FP’s visit to this facility ensured access to a clinician able to support the nursing staff trained in Nurse Initiated Management of ART (NIMART), by reviewing the patients not achieving virological suppression on the first-line ART regimes, patients on the second-line ART, as well as children on ART. Regional hospital specialists referred to the FP to ensure follow-up of patients with complex conditions, especially in the rheumatological, cardiac and neurological diagnostic categories.

The FP service enabled learning conversations, feedback and capacity building with junior doctors, registrars and other PHC colleagues. The service also helped to strengthen the relationships between the PHC team and the specialist teams at the referral hospital, as well as the tertiary subspecialist services in Cape Town. Importantly, this FP outreach service and consultant clinics at PHC facilities improved the patient care experience by improving access to specialised care, coordinated by the FP, closer to their home and community.

## Ongoing care coordination during a period of transition and the first year of a global pandemic

The FP also enabled informational continuity by maintaining an electronic database on his computer with key documents related to the patients. These included, for example, reports from the regional hospital, referral letters to specialists and results of special investigations. This central electronic repository was accessible to the senior family medicine registrar and the newly appointed FP, when the previous FP resigned in April 2020 to take on a new role. This ensured informational continuity of care during the transition period, as the senior family medicine registrar looked after the patients until the new FP started later in 2020.

In addition, as routine PHC services were de-escalated during the lockdown period from March to August 2020 of the coronavirus disease-2019 (COVID-19) pandemic, the FP outreach service to PHC was halted, and a list of patients for post-lockdown follow-up were kept. The referral hospital’s expectations of the FP also changed when their specialist outreach ceased during the COVID-19 pandemic. Consequently, the FP had to review patients previously seen during outreach visits from the regional hospital by their own specialists. The pandemic has resulted in the need for adjusting how the roles of the FP are implemented in practice.^5^

## Future directions

More detailed evaluation of the reasons for referral to the FP and the threshold for referral by different members of the PHC team may be needed.^6^ A larger scale analysis of the referral patterns to FP outreach services using more validated instruments with standardised diagnostic categories, such as the International Classification of Primary Care (ICPC),^7^ may provide useful information for policymakers and managers. A more nuanced understanding of the clinical skills and knowledge required by the newly qualified FP to manage patients referred by the PHC team will also assist postgraduate training programmes. Besides a model for registrars to ‘adopt’ a clinic and review patients weekly at the same PHC facility during their 4-year training, it would be good to also arrange for the registrar to join the FP during outreach visits to create opportunities for clinical governance (health service strengthening and capacity-building activities, such as reviewing of referrals and specialist item prescription practices) that will benefit both the registrar and clinic.

The phenomenon of a sole FP per subdistrict is not sustainable and limits the FP’s ability to strengthen the PHC service outside the district hospital, as the single FP needs to divide his or her contribution between the two. As part of the new human resources for health (HRH) strategy, there should be greater focus on creating more FP posts in the subdistricts to support high-quality, team-based PHC.^8,9^

## Conclusion

This short report sheds some light on the role of the FP as a consultant within the PHC team. This self-audit showed that a FP based at a rural district hospital can support the PHC teams in the subdistrict by providing easier access to more specialised services for ambulatory patients at PHC facilities. This enabled better care coordination between generalists and specialists by ensuring appropriate referrals and continuity of care for patients whose health conditions required close monitoring over time. More rigorous research is needed to further evaluate the role of the FP as a consultant within the PHC team.
